# ROS-Mediated Apoptosis and Genotoxicity Induced by Palladium Nanoparticles in Human Skin Malignant Melanoma Cells

**DOI:** 10.1155/2017/8439098

**Published:** 2017-07-16

**Authors:** Saud Alarifi, Daoud Ali, Saad Alkahtani, Rafa S. Almeer

**Affiliations:** Department of Zoology, College of Science, King Saud University, Riyadh, Saudi Arabia

## Abstract

The present work was designed to investigate the effect of palladium nanoparticles (PdNPs) on human skin malignant melanoma (A375) cells, for example, induction of apoptosis, cytotoxicity, and DNA damage. Diseases resulting from dermal exposure may have a significant impact on human health. There is a little study that has been reported on the toxic potential of PdNPs on A375. Cytotoxic potential of PdNPs (0, 5, 10, 20, and 40 *μ*g/ml) was measured by tetrazolium bromide (MTT assay) and NRU assay in A375 cells. PdNPs elicited concentration and time-dependent cytotoxicity, and longer exposure period induced more cytotoxicity as measured by MTT and NRU assay. The molecular mechanisms of cytotoxicity through cell cycle arrest and apoptosis were investigated by AO (acridine orange)/EtBr (ethidium bromide) stain and flow cytometry. PdNPs not only inhibit proliferation of A375 cells in a dose- and time-dependent model but also induce apoptosis and cell cycle arrest at G2/M phase (before 12 h) and S phase (after 24 h). The induction of oxidative stress in A375 cells treated with above concentration PdNPs for 24 and 48 h increased ROS level; on the other hand, glutathione level was declined. Apoptosis and DNA damage was significantly increased after treatment of PdNPs. Considering all results, PdNPs showed cytotoxicity and genotoxic effect in A375 cells.

## 1. Introduction

The establishment of nanotechnologies in the biomedical field has empowered uncountable applications for targeted drug delivery, detection, diagnosis, and imaging [1, 2]. Due to their size, structure variability, and optronics properties, metal nanoparticles are drawing interest for different biomedical applications [3, 4]. Palladium is a rare earth metal element and is released to the nature through anthropogenic activities. It is widely used in various fields of medicine, technology, and catalytic converters, and the incapability of nature to abolish this element has led to accumulation in milieu [5]. Ravindra et al. [6] have documented that palladium metal elements occur in airborne particles, roadside dust, sludge, and water, which ultimately accumulated in living organisms. Its concentration has been present in abiotic and biotic constituents of urban environments due to the capability of long-range transport of particles. Also, magnetic nanoparticles have specific bioapplications, such as magnetic hyperthermia, magnetic resonance imaging, target drug delivery, bacteria detection, enzyme immobilization, cell labeling, magnetic separation, and DNA enrichment [7]. Nowadays, palladium nanoparticles are used as a catalyst for environmental issues and in automobile industry similar as platinum metal [8, 9]. Exposure to palladium induced acute toxicity or hypersensitivity with respiratory symptoms and, frequently, contact dermatitis [10, 11]. Also, palladium is capable to exert a significant effect on the production and release of a number of cytokines [12, 13]. However, palladium levels have been found more near road dust and roadside soil [14, 15]. PdNPs have been found to have antimicrobial activities [16], and incubation of calf thymus DNA with palladium ions degraded DNA molecules [17].

Apoptosis is a programmed cell death activity, which plays specific role in chemotherapy against different cancer cells. It is a highly regulated process of cell death and activated by various stressors including cytokines, oxidative stress, and DNA damage [18, 19]. Pan et al. [20] reported that too much generation of reactive oxygen species (ROS) could encourage apoptosis, which had been useful to kill cancer cells. Fast production of ROS could activate apoptotic pathways. ROS could induce signaling pathways such as mitogen-activated protein kinase and the c-Jun-N-terminal kinase (JNK); a member of the MAPK family plays a crucial role in mitochondrial dysfunction and the subsequent initiation of apoptosis [21]. In this study, we explored the cytotoxic and DNA damaging potential of PdNPs on human skin malignant melanoma (A375) cells.

## 2. Materials and Methods

The cell culture medium (DMEM) was purchased from Gibco BRL (Grand Island, NY, USA). Neutral red (NR) dye, 3-(4,5-dimethyl-2-thiazolyl)-2,5-diphenyl-2H-tetrazolium bromide (MTT), penicillin, streptomycin, 2,7-dichlorodihydrofluorescein diacetate (H_2_-DCFDA), ethidium bromide (EtBr), and acridine orange (AO) were procured from Sigma-Aldrich (St. Louis, MO, USA). PdNPs (APS~15 nm) were obtained from US Research Nanomaterials Inc. All other common chemicals were purchased from markets.

### 2.1. Characterization

The nanoparticle suspensions were characterized by the following two methods.

#### 2.1.1. Transmission Electron Microscopy

PdNPs were suspended in water (40 *μ*g/ml) and dispersed by ultrasonification, and the carbon-copper grid was prepared and dried at room temperature before determining the size and shape of NPs. The determination of the size of PdNPs through TEM was done at 120 kV voltage (Model 1200 EX, JEOL Ltd., Tokyo, Japan).

#### 2.1.2. Dynamic Light Scattering (DLS)

The mean hydrodynamic size and zeta potential of suspended PdNPs in water and culture media were measured by DLS by using Zetasizer Nano-ZS equipped with 4.0 mW, 633 nm laser (Model ZEN3600, Malvern Instruments Ltd., Malvern, UK).

### 2.2. Cell Culture

A375 cells were procured from American Type Culture Collection (ATCC® CRL-1619™). Cells were subcultured in DMEM (Gibco) by adding 10% fetal bovine serum (FBS), penicillin (100 IU/ml), and streptomycin (100 *μ*g/ml) and incubated at 37°C in a CO_2_ incubator (5%).

Stock suspension of PdNPs (1 mg/ml) in DMEM (added with 10% FBS) was diluted to concentrations (5–40 *μ*g/ml) for morphology of cells, cytotoxicity, comet tests, generation of ROS, cell cycle arrest, and apoptosis. For each experiment, the suspension of PdNPs was freshly prepared, diluted to suitable doses, and instantly exposed to the cells. Culture medium without PdNPs served as the control in each experiment.

### 2.3. Morphology and TEM Ultrastructural Analysis of A375 Cells

After exposure to PdNPs, the morphology of A375 cells were seen by using an inverted microscope (Leica DMIL) for 24 and 48 h.

NPs treated and control cell were fixed in 2.5% glutaraldehyde/0.1 M PBS buffer pH 7.2 for one hour and then dehydrated through the graded series of acetone and embedded in Durcupan (Fluka Chemie, Buchs, Switzerland). The polymerization occurred after 24 hours at 65°C. The sections (2 *μ*m) of the cell were cut by using ultra microtome Reichert (Pabisch-Wien, Austria), stained with Toluidin blue 0.5% sodium carbonate, and observed under a light microscope. Ultrathin sections (~70 nm) were cut with a diamond blade, collected on slotted copper grills, colored with 3% uranyl acetate and lead citrate 6 [22], and observed by TEM (Model 1200 EX, JEOL Ltd., Tokyo, Japan) at an accelerating voltage of 80 kV.

### 2.4. Cytotoxicity Tests

#### 2.4.1. MTT Test

The effect of PdNPs (0, 5, 10, 20, and 40 *μ*g/ml) on cell viability was assessed by MTT assay [23]. Briefly, A375 cells was suspended in DMEM to 5 *×* 10^4^ cells per ml and aliquots (5 *×* 10^3^ cells/100 *μ*l/well) were put into each well of 96 multiplates. One day later, the media were changed with different concentrations of PdNPs and incubated for 24 and 48 h in a CO_2_ incubator. After exposure, the supernatant was removed and a 100 *μ*l MTT solution (5 mg/ml in medium) was added per well and incubated for 4 hours in CO_2_ incubator. After incubation, the MTT solution was discarded and formed crystal was dissolved in DMSO (100 *μ*l). The optical density of each well was determined at 540 nm.

#### 2.4.2. Neutral Red Uptake (NRU) Assay

The NRU assay was done according to Borenfreund and Puerner [24] methods. The cells were grown in 96-well plates and treated with different concentrations of PdNPs for 24 and 48 h. After the treatment of PdNPs for 24 and 48 h, the suspension was discarded from plates and the cells were washed with PBS, and NRU test was done for cell viability. After adding neutral red (50 mg/ml in DMEM), the culture plates were incubated for 4 h in CO_2_ incubator. After incubation, the cells were fixed through fixative solution (1% CaCl_2_ and 0.5% formaldehyde) and dye was extracted with a mixture of acetic acid, ethanol, and water (1 : 50 : 49). NRU was observed at 540 nm, and it was expressed as a percentage (%) of uptake by untreated control cells.

### 2.5. Quantification of Intracellular Reactive Oxygen Species (ROS) Assay

ROS generation was measured by using ROS Assay Kit (Cell Biolabs Inc., San Diego CA, USA). A375 cells (1.10^4^ cells/well) were cultured in 96-well black plates. After incubation with various doses of PdNPs for 24 and 48 h, cells were rinsed with HBSS. DCFH-DA (10 *μ*M) was added to the cells at 37°C for 1 h in the dark. Nonfluorescent DCFH-DA is changed to fluorescent DCF in ratio to the quantity of ROS generation in cells. The fluorescence signal was determined by using a spectrofluorometer (Beckman Pasadena, CA, USA) at excitation (485 nm) and emission (530 nm) wavelengths.

### 2.6. Intracellular ROS Detection by Fluorescence Microscopy

ROS production was checked by using the above ROS assay kit. The cells (3.10^5^ cells/well) were cultured on coverslips in 6-well plates. After exposure to PdNPs for 24 and 48 h, cells were rinsed with PBS and cells were incubated with DCFH-DA (10 *μ*M) at 37°C for 1 h in the dark. Nonfluorescence DCFH-DA is changed to fluorescent DCF in ratio to the quantity of ROS production in the cells. The cells were mounted, and fluorescent images were taken by using a fluorescence microscope (BZ-9000 Keyence, Osaka, Japan) at 485 and 530 nm wavelengths.

### 2.7. Assessment of Oxidative Stress

In addition, in analysis of ROS generation and cytotoxicity, MDA (malondialdehyde) content was determined as the final product of lipid peroxidation. The defence system against free radical attack was observed by the assessing of superoxide dismutase (SOD) activity. After treatment of PdNPs (0, 5, 10, 20, and 40 *μ*g/ml) for 24 and 48 h, PdNPs were washed with chilled PBS and lysed in chilled RIPA buffer (phenylmethylsulfonyl fluoride and phosphatase inhibitor) for 30 min. Lysate of the A375 cell was centrifuged at 12000 rpm for 10 min at 4°C, and supernatant was collected for determination of MDA production, GSH, and SOD activity. All observations were done with Cayman chemical kit (Ann Arbor, MI, USA) according to the manufacturer's instructions. The concentration of protein of cell lysate was quantified by using bicinchoninic acid (BCA) protein assay (Sigma-Aldrich).

### 2.8. Chromosome Condensation and Caspase-3 Activity Assays

DAPI (4′,6-diamidino-2-phenylindole) dyes were used to observe the apoptosis, including chromosome condensation. A375 cells (3 × 10^3^ cells per well) were cultured in plates (6-well). After overnight incubation, PdNPs (20 and 40 *μ*g/ml) were added for 24 and 48 h. After treatment for fixed periods, the cells were stained with DAPI (10 *μ*g/ml) for 30 min at room temperature. Two hundred cell images were captured by inverted fluorescence microscope (magnification of 40x, Nikon Eclipse 80i).

The level of caspase-3 enzyme was quantified from the cleavage of the caspase-3 substrate I (N-acetyl-DEVD-p-nitroaniline). The p-nitroaniline was used as the standard. Cleavage of the substrate was observed at 405 nm, and it was expressed in pmole of the product (nitro aniline) per min/mg of protein.

### 2.9. AO and EtBr Staining and Caspase-3 Activity Assays

AO and EtBr dyes were used to observe the apoptosis, including chromosome condensation. A375 cells (3000 cells per well) were cultured in plates (6 wells). After overnight incubation, PdNPs (20 and 40 *μ*g/ml) were added for 24 and 48 h. After treatment for fixed periods, the cells were stained with AO (10 *μ*g/ml) and EtBr (10 *μ*g/ml) for 30 min at room temperature. Two hundred cell images were captured by inverted fluorescence microscope (magnification of 40x, Nikon Eclipse 80i).

### 2.10. Cell Cycle

A375 cells were cultured in six-well culture plates to 30% confluence and then exposed for 24 h with PdNPs (20 and 40 *μ*g/ml). The cells were collected and fixed in ice-cold ethanol (70%) on ice for 30 min with regular mixing. The cells were rinsed with chilled PBS and resuspended in 500 *μ*l of 0.8% PBS containing 0.1% Triton X-100 and 0.1 mg/ml of DNase-free RNase for 5 h at 37°C. Later, the digestion was finished; 5 *μ*l of ethidium bromide (2 *μ*g/ml) solution was added, and the cells were incubated for 1 h at 37°C. After incubation, the cells were rinsed two times with PBS, a histogram of cell distribution was obtained using the FL2 channel (595 nm) of a flow cytometer (FACS, BD Biosciences, Franklin Lakes, NJ, USA), and the distribution of cells in the different cell cycle phases was analyzed, using the CellQuest Pro software (BD Biosciences), from the histogram generated.

### 2.11. Comet Test

A375 cells (5.10^4^ cells/well) were seeded in 6-well plates and exposed to different concentrations of PdNPs for 24 and 48 h. Comet test was done as a three-layer procedure [25]. Cells were harvested by trypsinization and resuspended in culture medium, and cell suspension was centrifuged at 1000 rpm at 4°C for 5 min. The cell pellet was dissolved in ice cold PBS for comet test. The parameters used to measure DNA damage in cells were % tail DNA and olive tail moment (OTM). Images from 50 random cells (25 from each replicate slide) were analyzed for each experiment.

### 2.12. Statistical Analysis

The data are examined by using one-way ANOVA, and results were presented as average ± SE. A *p* value < 0.05 and 0.01 was considered statistically significant and highly significant, respectively. The data were analyzed with the Statistical Package for Social Sciences (SPSS, Chicago, IL).

## 3. Results

### 3.1. Characterization of Palladium NPs

**Figure 1:**
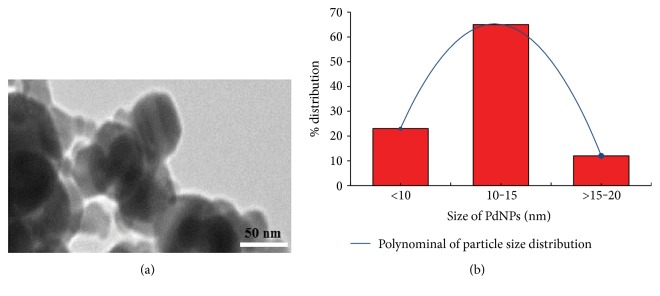
Characterization of PdNPs. (a) TEM image. (b) Size distribution (%) of PdNPs generated by TEM image.

The average hydrodynamic diameter of PdNPs in Milli Q water as determined by DLS was 12 nm, and zeta potential was −7.8 mV. The characterization of PdNPs was also performed in DMEM added with FBS (10%). In DMEM, nanoparticles showed a few increase in the hydrodynamic size (16.50 nm) with a parallel reduction in the zeta potential (−6.3 mV). The mean particle size of the PdNPs was spherical with mean diameter 14.70 ± 2.30 nm (Figure 1).

### 3.2. Alteration in Morphology, Cellular Uptake

**Figure 2:**
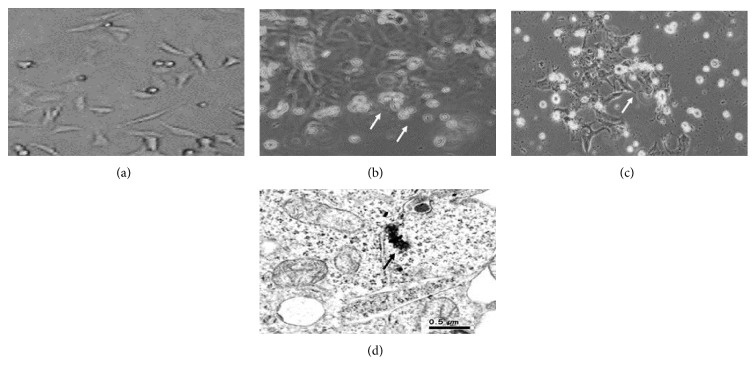
Morphology of human skin malignant melanoma (A375) cells. (a) Control. (b) At 40 *μ*g/ml PdNPs for 24 hours. (c) At 40 *μ*g/ml PdNPs for 48 hours. (d) TEM micrographs of PdNP internalization. Different types of cytoplasmic vesicles were observed. White arrow = damaged cells and black arrow = small NP aggregates.

After treatment of PdNPs for 24 and 48 h, the shape of A375 cells was observed under an inverted microscope (Innovation Way Carlsbad, CA 92009). It was deformed into a round shape (Figures 2(b) and 2(c)) as compared to control cells (Figure 2(a)). PdNPs enter into the cell organelles and made clusters of NPs of some hundreds of nm in diameter. Different types of cytoplasmic vesicles were observed (Figure 2(d)).

### 3.3. Cell Toxicity

**Figure 3:**
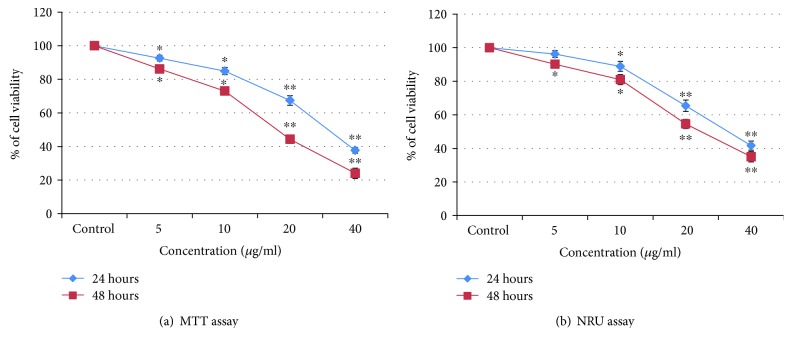
Percentage cell viability due to exposure of PdNPs to A375 cells for 24 and 48 hours, as assessed by (a) MTT and (b) NRU assays. Each value represents the mean ± SE of three experiments. ^∗^*p* < 0.05 and ^∗∗^*p* < 0.01 versus control.

Determination of A375 cells viability was performed by MTT and NRU assays (Figure 3). PdNPs induced a significant (*p* < 0.05, 0.01) cytotoxic effect in A375 cells. There is a significant reduction in cell viability of A375 cells according to a dose- and time-dependent manner. The MTT data indicated a 62.3% and 75.94% decrease (compared to the control) while the NR uptake was decreased to 58% and 65.01% at 40 *μ*g/ml, respectively, indicating a cytotoxic potential of PdNPs for 24 and 48 h (Figure 3).

### 3.4. ROS Generation and Oxidative Stress

**Figure 4:**
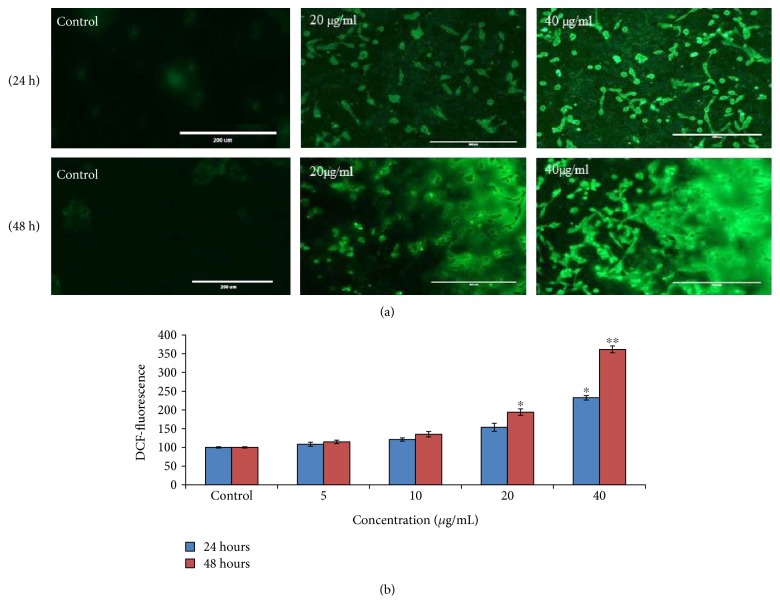
ROS production induced by PdNPs. (a) The fluorescence image of A375 cells treated with 20–40 *μ*g/ml of PdNPs for 24–48 h and stained with DCFHDA. Bar = 400 *μ*m. (b) % ROS production due to PdNPs in cells. Images were snapped in phase contrast cum fluorescence microscope (Nikon, model 80i). Each value represents the mean ± SE of three experiments. ^∗^*p* < 0.05 and ^∗∗^*p* < 0.01 versus control.

To find out the production of ROS after exposure of PdNPs, we have measured it by H2-DCFDA-loaded cells by fluorescence microplate reader and fluorescence inverted microscope. Results indicated that cells exposed to PdNPs (5, 10, 20, and 40 *μ*g/ml) significantly increased ROS generation (Figure 4).

**Figure 5:**
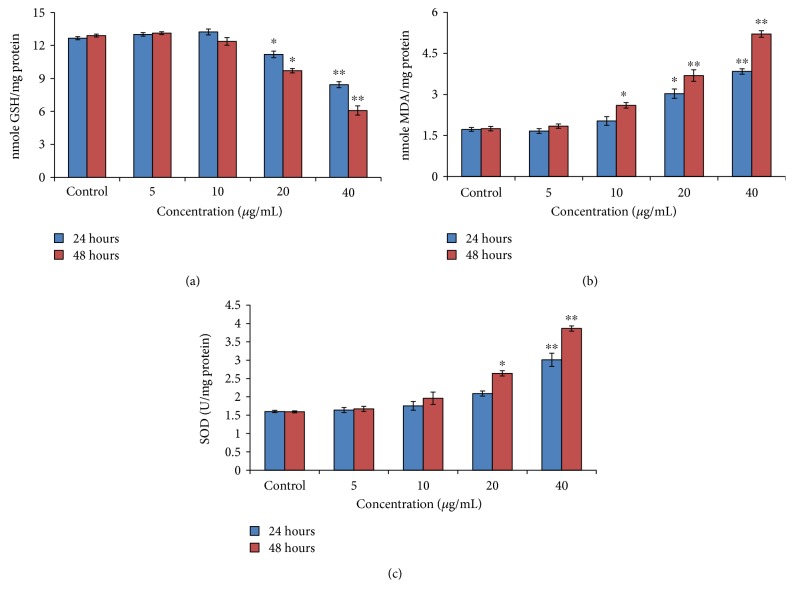
PdNPs induced oxidative stress biomarkers. (a) GSH. (b) LPO. (c) SOD in A375 cells. Each value represents the mean ± SE of three experiments. ^∗^*p* < 0.05 and ^∗∗^*p* < 0.01 versus control.

Figure 5(a) shows that PdNPs significantly reduced glutathione level at a higher concentration of nanoparticles, and LPO, superoxide dismutase, and catalase activities were increased according to a time- and concentration-dependent manner (Figures 5(b) and 5(c)).

A highly significant reduction (*p* < 0.01) in cellular GSH content (15.76% and 49.73%) was observed in A375 cells at 40 *μ*g/ml concentration after 24 h and 48 h exposure to PdNPs, respectively (Figure 5(a)).

### 3.5. Chromosome Condensation and Caspase-3 Activity

**Figure 6:**
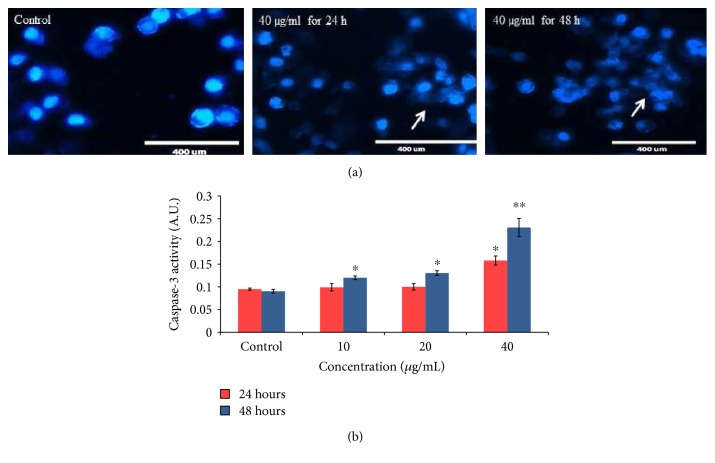
(a) Chromosomal condensation at 40 *μ*g/ml for 24 and 48 h and (b) induction of caspase-3 activity in A375 cells after exposure to PdNPs for 24 and 48 h. Each value represents the mean ± SE of three experiments. ^∗^*p* < 0.05 and ^∗∗^*p* < 0.01 versus control. Arrow (→) indicates fragmented chromosome.

We have measured morphological change in exposed cells by using DAPI labeling. After staining through DAPI dye, the cells' image was captured by fluorescence microscope and more fragmented nuclear materials are found at higher concentration of PdNPs (Figure 6(a)). The activity of caspase-3 was increased in a dose- and time-dependent manner (Figure 6(b)).

### 3.6. AO/EtBr Staining

**Figure 7:**
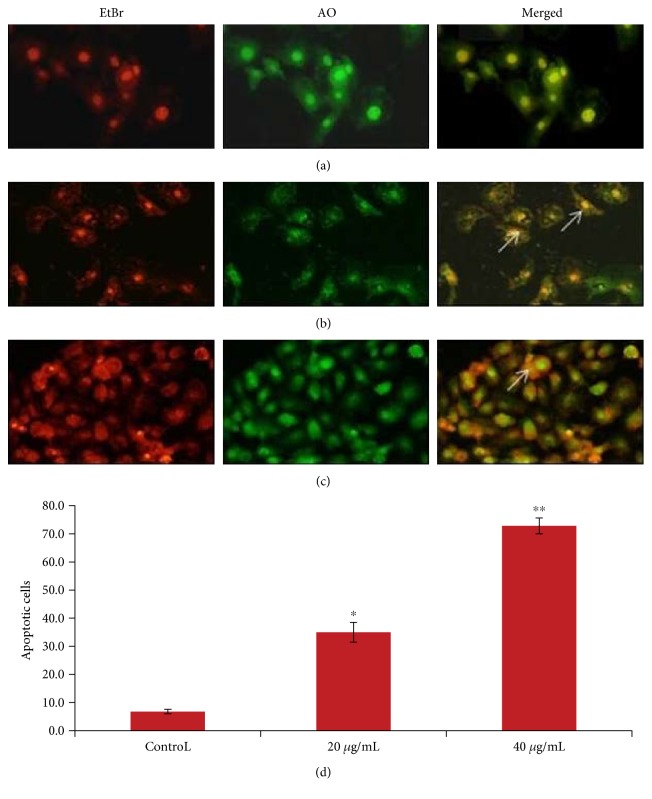
PdNPs induced apoptotic morphological changes. Fluorescence microphotograph of A375 cells showed apoptotic morphological changes in nanoparticle-treated cells: (a) control, (b) 20 *μ*g/ml, (c) 40 *μ*g/ml PdNP-treated cells, and (d) percentage apoptotic cells were calculated by scoring apoptotic (EtBr cells) and viable (AO cells) in 200 A375 cells. Values are given as means ± SE of triplicate experiments in each group. Significance at ^∗^*p* < 0.05 and ^∗∗^*p* < 0.01 versus control. Arrow marks (→) represent the apoptotic indices.

The effect of PdNPs showed enhanced apoptosis which was measured by AO/EtBr morphological assays in A375 cells (Figure 7). The assay is based on nuclear morphology, specific for apoptosis. The live cells showed normal nuclear chromatin with green fluorescence, but apoptotic cells contain fragmented DNA (intense orange color). PdNP treatment illustrated significant number of apoptotic cells as compared to the control (Figure 7).

### 3.7. Analysis of Cell Cycle

**Figure 8:**
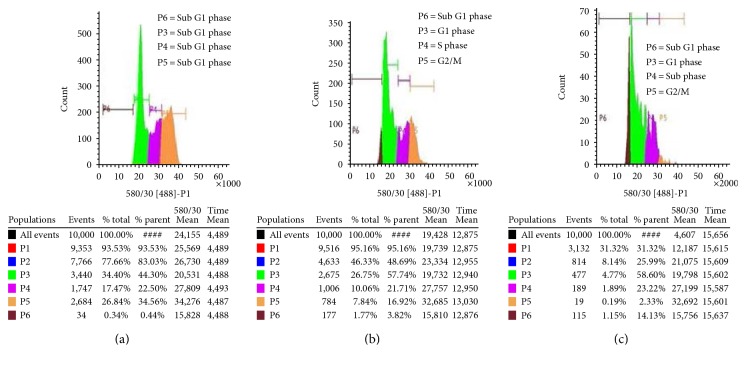
PdNPs enhance cell cycle arrest and appearance of sub-G1 phase. A375 cells were treated with PdNPs (20 *μ*g/ml and 40 *μ*g/ml and incubated for 24 h). (a) Control, (b) 20 *μ*g/ml, and (c) 40 *μ*g/ml PdNPs.

Cell cycle phase analysis was done by using flow cytometry, to determine PdNP effects on cell cycle kinetics. Results indicated that more cells are present in sub-G1 phase of cell cycle as compared to the untreated group; however, the % of cells in G2 phase was decreased progressively in a dose-dependent manner. The quantity of cells in sub-G1 stage increased from 0.78% (untreated group) to 5.59% and 15.28%, at 20 *μ*g/ml and 40 *μ*g/ml concentrations, respectively (Figure 8).

### 3.8. DNA Fragmentation

**Figure 9:**
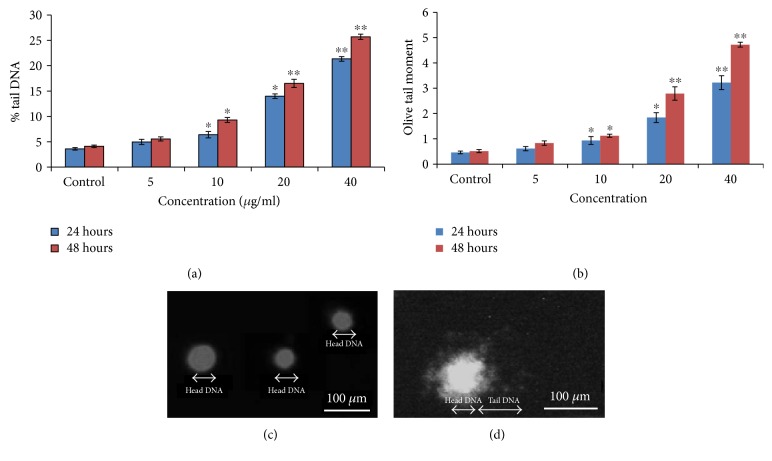
DNA strand breakage in A375 cells due to PdNPs: (a) % tail DNA, (b) olive tail moment, (c) control cell, and (d) exposed cell to PdNPs (40 *μ*g/ml) for 48 hours. Each value represents the mean ± SE of three experiments. ^∗^*p* < 0.05 and ^∗∗^*p* < 0.01 versus control.

DNA damage was significantly increased in PdNP-treated cells as compared to the control as marked by the Comet test parameters, namely, tail DNA (%) and olive tail moment (OTM), respectively, at 5, 10, 20, and 40 *μ*g/ml (Figure 9). Furthermore, for each treatment, a significant increase in the damage scores was observed with the increase of the exposure time (Figure 9).

## 4. Discussion

The current experiment reveals the toxicity of PdNPs on human skin malignant melanoma (A375) cells and delivers an important understanding into the probable mechanism by which PdNPs induce its toxic effects on skin cells. Few studies are available on the long-term effects of palladium exposure, but there is no available data on underlying molecular mechanisms. The present results indicate the cytotoxic and genotoxic effects of PdNPs in A375 cells. Our results also revealed that the mode of cell death was apoptosis which was mediated by the ROS-triggered cleavage of caspase-3. PdNPs tend to aggregate both in water and in culture media, and therefore, the interaction between relatively strongly bonded aggregates or soft agglomerates of NPs containing metals and live cells may be a key passage in justifying NP toxicity. Single nanoparticles or few aggregates (100 nm) may enter by passive diffusion and localize in the cytoplasm and other cell organelles. The examination of ultrasection of treated cells demonstrates that most of NPs (agglomerates of different size) passed into the cells by endocytosis. Result of uptake determination revealed that PdNPs accumulated in A375 cells.

We have used human diploid immortalized skin malignant melanoma (A375) cells which represent one of the widely used cellular models for toxicological assay. However, some research have reported that internalization of NPs reduced the proliferative capacity of the cells at long time exposure [26]. PdNPs may exhibit a variety of effects such as cytotoxicity, generation of intracellular ROS, and DNA damage. To support the results of the MTT and NRU assays, we observed the effect of PdNPs on morphology of cell. The cells were exposed to PdNP concentration at which it induced significant toxicity. Our observations indicate that PdNPs (at 40 *μ*g/ml) made significant morphological alterations, which were more significant with increasing exposure time and concentrations of PdNPs. As well, the cells showed shrunken cell membranes and debris was seen in the A375 cells exposed with 40 *μ*g/ml for 24 and 48 h, demonstrating that the morphology of cells significantly vary from that of the unexposed sample. In addition, the A375 cell was susceptible to PdNPs, which changed the viability and shape. The density of cell was more reduced in the PdNP-exposed cell control. PdNPs were able to deter in a concentration and time-dependent manner cell growth and viability. Inhibition of cell growth was specifically related with a reduction of the percentage of the G0/G1 phase of the cell cycle and an accumulation of cells in the S and G2/M phases of the cell cycle.

The increase in oxidative stress and apoptotic cells confirmed the acuteness of PdNP toxicity. Excessive ROS production causes harmful oxidative stress, which is implicated in many chronic diseases involving inflammatory processes. We observed that PdNPs have capability to induce oxidative stress with formation of ROS and induction of DNA damage as measured by comet test. However, it has to be underlined that even a minor effect observed in vitro might be related to important biological consequences in vivo especially in the case of exposition acting for a long period of time as it might occur in chronically exposed populations. In fact, ROS can attack biomolecules and induce genotoxic damage, cell dysfunction, and cell death [27]. Iavicoli et al. [28] have reported that PdNPs caused a significant increase in DNA fragmentation in female Wistar rats. Hu et al. [29] have reported that cell toxicity induced by ROS accompanies an increase in lipid peroxides. The plasma membrane oxidation, one of the initial processes in oxidative damage, may be measured by quantification of malondialdehyde, a final byproduct of lipid peroxidation. Regular treatment of cells to free radicals induced defense mechanisms involving enzymatic and nonenzymatic antioxidants. Many antioxidant enzymes show a significant role in the detoxification process to prevent cell damage caused by oxidative stress and other types of stress caused by exogenous factors such as NPs [30]. So, we examined the function of PdNPs' antioxidative or oxidative roles by treating cells with various doses and exposure time and evaluating both MDA and antioxidant enzymes. Exposure of A375 cells with different doses of PdNPs (0–40 *μ*g/ml) significantly increased the intracellular MDA level, indicating that PdNPs may potentially induce oxidative damage in cells. In addition, PdNPs increased the MDA level dose and time dependently. To our knowledge, it is the first time to study the toxic mechanism of PdNPs in A375 cells. Results show that PdNPs affect cell growth and cell cycle progression in A375 cells and suggest the hypothesis that the induction of apoptosis is correlated to a chunk of cell cycle progression at the G2/M phase. On the basis of these findings, it is not confirmed that cell cycle protein is a result of direct/indirect (i.e., through the intracellular accumulation of ROS and/or DNA damage) action of PdNPs. PdNPs elicit genotoxic effects through direct interaction with DNA or indirectly via NP-induced oxidative stress and apoptotic responses. Caspases are essential modules of the pathways of cell death, and caspase-3 is triggered by several death signals and cleaves a variety of cellular proteins, which are essential for DNA damage and morphological alterations in cells undergoing apoptosis [31]. DNA damage is one of the most important final and irreversible events in apoptosis [32]. Also, fragmentation of DNA is a hallmark of apoptosis. So, we find out whether PdNP-induced activation of caspase-3 is involved in DNA damage.

A375 cells were treated with PdNPs for 24 and 48 h and then cells were stained with AO/EtBr and DAPI. The observations demonstrate that exposure to PdNPs induced a significant number of fragmented chromosome cells, whereas no apoptotic cells were observed in the controls. Thus, our observations in the present study are corroborated with the finding of Takaki et al. [33] in leukemia L1210 cells for TiO_2_NPs. DNA damage after exposure to PdNPs for 24 and 48 h is dependable with an increase in caspase-3 activity after PdNP exposure for 24 and 48 h. Our findings suggest that PdNPs induce cytotoxicity via fragmentation of DNA and activation of caspase-3; however, the degree of cytotoxicity is dependent on the physical properties of NPs. On the basis of these findings, it can be concluded that PdNPs have toxic effects in A375 cells. These initial findings indicate the need to carry out further investigations to identify the different molecular mechanisms of PdNP toxicity that could be useful to define the risk assessment and human health.
